# No one left behind: how Colombia is adapting its trachoma programme to reach indigenous populations

**Published:** 2022-12-16

**Authors:** Julian Trujillo-Trujillo, Martha Saboya, Tim Jesudason

**Affiliations:** 1 Integrated Management of Emerging, Re-emerging and Neglected Diseases: Ministry of Health and Social Protection, Bogotá, Colombia.; 2 Neglected Infectious Diseases (NID) Epidemiology Advisor: Pan-American Health Organization, Washington DC, USA.; 3 Special Projects and Campaign Partnerships: International Coalition for Trachoma Control, London, UK.

**People-centred and human rights-based approaches increase access to eye care.**

**Figure:**
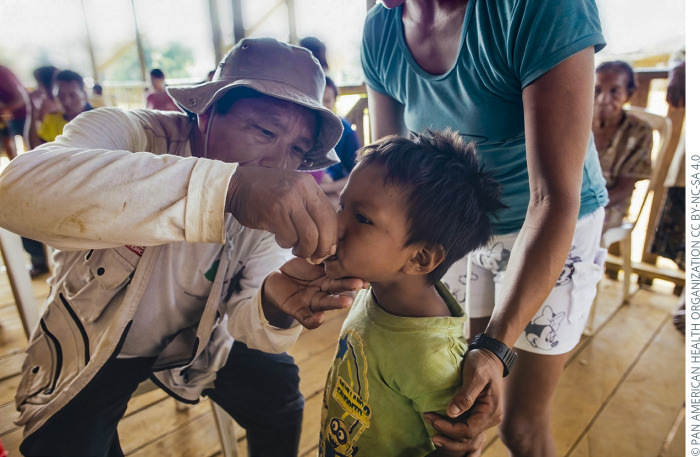
Mass drug administration for trachoma. **COLOMBIA**

Indigenous and nomadic communities vary significantly in terms of their cultural practices, spoken languages, and socioeconomic and political status. However, many indigenous communities share common challenges in accessing eye health services, including interventions for trachoma, the world’s leading infectious cause of blindness.

In Colombia, some nomadic indigenous groups, including the Jupdá makú, Yujup makú, and Cacua makú, live in the Amazon basin their ancestral territory, where they travel through the jungle in seasonal cycles. They have an increased risk of trachoma due to various geographical, socioeconomic, and cultural risk factors, including insufficient access to basic services to prevent trachoma, such as health education, safe water, and sanitation, and limited access to health care services.

Traditional beliefs and practices can also interfere with the delivery of eye care. For example, many indigenous communities believe that trachomatous trichiasis (TT), the late blinding stage of trachoma, is caused by eating *mojojoy* (the larvae of palm beetles). These communities believe that the hairs of the larvae grow inside the eyelid, which then damages the cornea of the eye. In other communities, traditional healers use a resin from the caraña tree as a wax to remove eyelashes. This form of epilation offers relief from the pain of eyelashes scraping against the cornea, but can cause skin lacerations.

To address these and other challenges, Colombia’s Ministry of Health and Social Protection decided to include indigenous communities in the planning and implementation of its national trachoma programme from 2011–2018. The ministry held annual meetings, bringing together programme teams and indigenous communities to identify goals, build trust, and improve knowledge about trachoma. In the Vaupés region, for example, local authorities, with the guidance of the Ministry of Health and Social Protection, worked with leaders, traditional authorities, and communities to identify nomadic people and those living in settlements who had TT, to provide health education, and to distribute antibiotics.

People with TT were offered a transfer to Mitú, the capital of Vaupés, for assessment and corrective treatment. This was carried out by specialists using the bilamellar tarsal rotation technique. All transfers, accommodation, food, and medical treatment were provided by the health authorities with support from the Pan American Health Organization (PAHO).

Throughout the programme, efforts were made to ensure that interventions were culturally appropriate. Health workers collaborated with the traditional healers who perform rituals that are important to the indigenous communities; this helped to increase participation in the trachoma programme. To increase the efficiency of the programme, health workers also provided education and treatment for other health issues, such as soil-transmitted helminths. 

Colombia’s people-centred and rights-based approach to health care has increased access and participation in health services for indigenous communities. The programme has resumed activities suspended during the COVID-19 pandemic. Research is being planned that will generate evidence about access, barriers, and the role of trainers in delivering trachoma programmes in the Amazon region.

Colombia adheres to the Regional Policy on Ethnicity and Health, adopted by PAHO Member States in September 2017, which commits it to developing inclusive and collaborative solutions, adapted to the social, cultural, and geographic context, and to address health gaps, including interventions for neglected tropical diseases and eye health, in indigenous communities. Going forward, the national trachoma programme requires increased involvement by all stakeholders, support and funding to provide health services to those communities, and improvements in the access to (and use of) water and sanitation to achieve health targets, including the elimination of trachoma as a public health problem.

